# Weight loss decreases progressive left ventricular remodeling: The Multi-Ethnic Study of Atherosclerosis

**DOI:** 10.1186/1532-429X-16-S1-O2

**Published:** 2014-01-16

**Authors:** Siddique A Abbasi, Ravi V Shah, Venkatesh L Murthy, John Eng, Colin Wu, Pamela Ouyang, Raymond Y Kwong, Allison Goldfine, David Bluemke, Joao A Lima, Michael Jerosch-Herold

**Affiliations:** 1 Section of Non-invasive Cardiovascular Imaging (Department of Internal Medicine/Division of Cardiology and Department of Radiology), Brigham and Women's Hospital/Harvard Medical School, Boston, Massachusetts, USA; 2 Department of Internal Medicine (Division of Cardiology), Massachusetts General Hospital, Boston, Massachusetts, USA; 3 Department of Medicine (Cardiovascular Medicine Division) and Department of Radiology (Nuclear Medicine and Cardiothoracic Imaging Divisions), University of Michigan, Ann Arbor, Michigan, USA; 4 Office of Biostatistics Research, National Heart, Lung, and Blood Institute, Bethesda, Maryland, USA; 5 Department of Internal Medicine, Division of Cardiology, Johns Hopkins Bayview Medical Center, Baltimore, Maryland, USA; 6 Department of Internal Medicine/Division of Endocrinology, Joslin Diabetes Center/Brigham and Women's Hospital, Boston, Massachusetts, USA; 7 Radiology and Imaging Sciences, National Institutes of Health Clinical Center, National Institute of Biomedical Imaging and Bioengineering, Bethesda, Maryland, USA; 8 Department of Radiology, Brigham and Women's Hospital, Boston, Massachusetts, USA

## Background

Obesity (body mass index, BMI > 30 kg/m2) is an independent risk factor for incident heart failure (HF). Effects of weight change on cardiac structure have not been extensively investigated in large community-based populations. We hypothesized that weight loss and gain in the Multi-Ethnic Study of Atherosclerosis (MESA) would coincide with changes in left ventricular (LV) mass -- key features in the progression to obesity-related HF.

## Methods

To investigate the association of longitudinal changes in weight on ventricular remodeling, we investigated 2,351 patients in MESA who underwent two serial cardiac magnetic resonance imaging (CMR) examinations at initial enrollment (2002) and at follow-up (2011) with available obesity status. Canonical parameters of LV structure and function (height-indexed LV mass, LV volumes, and LV ejection fraction) were measured. MESA participants were classified by obesity status (normal weight: 18.5-25 kg/m2; overweight/obese ≥ 25 kg/m2). We constructed splines for linear and logistic models using generalized additive models to assess the form of the continuous relationship between indexed LV mass changes and weight change qualitatively. Next, we constructed multivariable linear models, adjusted for confounders involved in the pathogenesis of LV hypertrophy that could be altered by weight change, including: glycemic status, hypertension, waist-to-hip ratio, body-mass-index, and biomarkers of inflammation. Finally, the multivariable linear model was adjusted for age, gender, race, income, educational status, smoking, Exam 1 BMI, and height-indexed LV mass at Exam 1.

## Results

Of the overall cohort studied, 257 individuals (11%) experienced ≥10% weight loss (median 10.2 kilograms) and 194 (8%) had ≥10% weight gain (median 10.0 kilograms). After adjustment for hypertension, diabetes, age, race, and other clinical risk factors, every 10% decrease in weight was associated with a fully covariate-adjusted 3% additional decrease in height-indexed left ventricular mass. Every 10% loss in body weight increased the odds of a 10% or greater drop in left ventricular mass by 50%. Finally, regression models suggested linear decreases in left ventricular mass regression with increasing weight loss, suggesting no threshold effect for weight loss on cardiac remodeling.

## Conclusions

Weight loss is associated with significant beneficial effects on cardiac remodeling, even after adjustment for baseline obesity-related cardiometabolic risk. There is no threshold for the weight change needed before benefits on cardiac occur, suggesting that any degree of weight loss may be beneficial to the heart.

## Funding

MESA was supported by contracts NO1-HC-95159 through N01-HC-95169 from the National Heart, Lung, and Blood Institute. Dr. Abbasi is supported by a T32 fellowship. Dr. Shah is supported by an American Heart Association Post-Doctoral Fellowship Award (11POST000002) and a training grant from the Heart Failure National Institutes of Health Clinical Research Network (U01-HL084877). Dr. Jerosch-Herold receives support through R01-HL-65580. All other authors have no financial disclosures relevant to the content of this manuscript.

**Table 1:** Linear regression for percent change in height-indexed LV mass between Exam 1 and Exam 5

Parameter	Linear Model		
	Estimate	95% Confidence Interval	P-value
Age, y	-0.05	(-0.12 to 0.02)	0.18
Female gender	-7.67	(-8.93 to -6.4)	< 0.0001
Race			
White	referent	-	-
Chinese	0.11	(-1.67 to 1.88)	0.9
African-American	2.83	(1.42 to 4.23)	< 0.0001
Hispanic	-0.42	(-2.04 to 1.19)	0.61
Smoking history (ever smoker)	-0.68	(-1.76 to 0.39)	0.21
Income	-0.13	(-0.33 to 0.06)	0.19
Education	-0.04	(-0.31 to 0.23)	0.77
BMI at Exam 1 (per log), kg/m2	24.51	(17.67 to 31.35)	< 0.0001
Glycemia			
Normal	referent	-	-
IFG	-0.75	(-2.49 to 0.99)	0.4
Untreated DM	-2.56	(-6.5 to 1.38)	0.2
Treated DM	0.92	(-1.29 to 3.12)	0.42
Δ Glycemia	-0.13	(-0.83 to 0.58)	0.72
Hypertension stage	2.73	(2.19 to 3.28)	< 0.0001
Δ Hypertension stage	2.88	(2.43 to 3.33)	< 0.0001
Number of antihypertensive classes at Exam 1	0.2	(-0.43 to 0.83)	0.54
C-reactive protein (per log)	-0.46	(-0.98 to 0.07)	0.09
Waist Circumference at Exam 1 (per cm)	0.07	(-0.02 to 0.15)	0.11
LVMI at Exam 1 (g/m2.7)	-0.8	(-0.88 to -0.73)	< 0.0001
Weight change from Exam 1 to 5 (per 10% decrease)	-2.76	(-3.42 to -2.1)	< 0.0001

**Figure 1:**
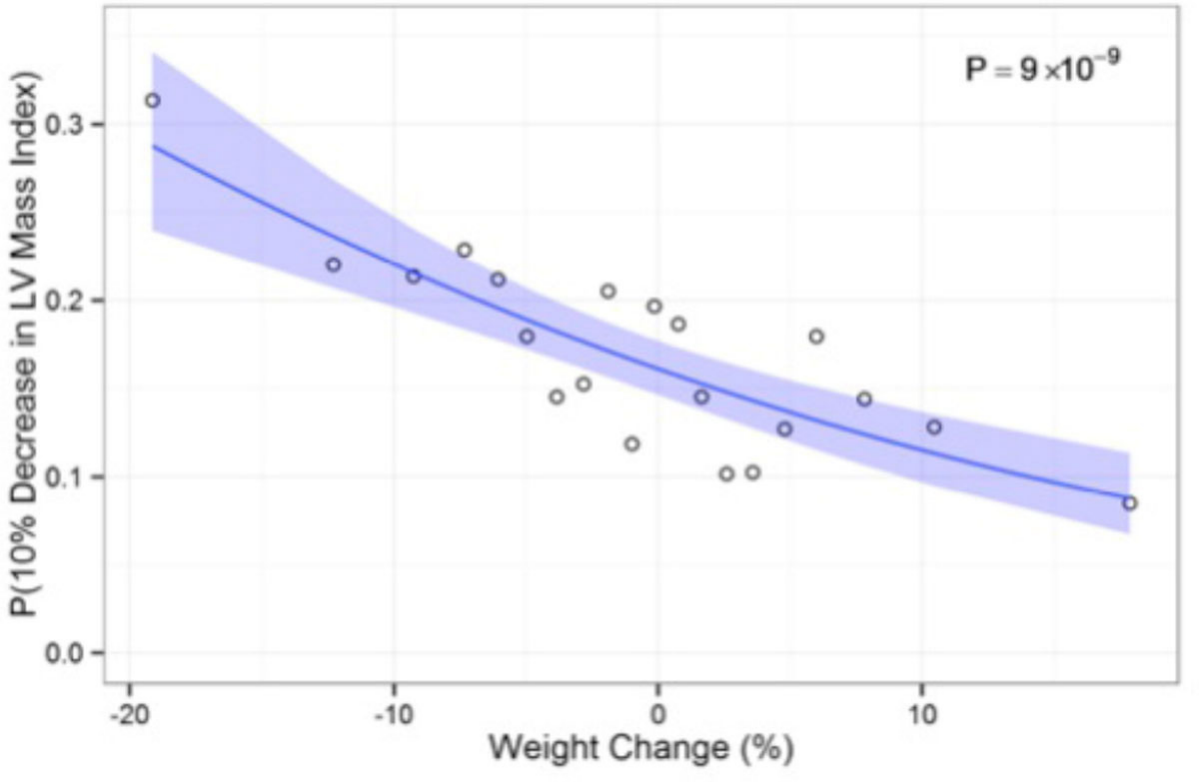
**Probability of significant regression in LV mass as a function of weight loss**. The probability of a significant regression (defined as ≥10% decrease) in height-indexed LV mass increases with greater degrees of weight loss. Data from all participants was grouped into twenty quantiles according to percent body weight change between Exam 1 and 5. The proportion of individuals in each quantile experiencing a 10% or greater reduction in height-indexed LV mass is plotted versus the mean percentage body weight change for that quantile. The underlying data were fit to a logistic model.

